# Occupational safety in coal logistics: a systematic review of systemic risks and human factors

**DOI:** 10.3389/fpubh.2026.1824250

**Published:** 2026-04-23

**Authors:** W. Prima Addini-Alia W. Abdullah, Kadir Arifin, Mohammad Lui Juhari, Kadaruddin Aiyub, Azlan Abas

**Affiliations:** Centre for Research in Development, Social and Environment (SEEDS), Faculty of Social Sciences and Humanities, Universiti Kebangsaan Malaysia, Bangi, Malaysia

**Keywords:** coal logistics, human factors, occupational safety, PRISMA 2020, risk management, systemic risk

## Abstract

**Background:**

Occupational safety research in the coal industry has traditionally prioritized extraction hazards. Consequently, the complex, interconnected risk landscape of the post-extraction supply chain, encompassing transportation, handling, and storage, remains fragmented. Addressing this gap requires a shift from isolated risk assessments to a more holistic approach. This study adopts a systemic perspective to systematically identify occupational accident risks and synthesize risk management practices specifically within coal logistics operations.

**Methods:**

Guided by the PRISMA 2020 framework, a systematic review of 68 peer-reviewed articles published between 2004 and 2025 was conducted to map these systemic interdependencies.

**Results:**

Thematic analysis indicates that ‘Human and Organizational Risk Factors’ constitute the predominant hazard category (51%), followed by ‘Mechanical and Operational Failures’ (31%) and ‘Environmental and Geological Hazards’ (18%). Crucially, a systemic synthesis of the findings challenges the conventional linear attribution of accidents to individual negligence. Results demonstrate that recurrent ‘unsafe acts’ are frequently symptoms of deeper systemic deficiencies within the socio-technical environment, including excessive workload and psychosocial stress. Furthermore, the review identifies a significant geographical concentration of research in China (66%), which influences the dominant risk management narrative toward top-down safety management systems and state-led intelligent automation.

**Conclusion:**

While technical hazards such as conveyor deviation and spontaneous combustion are universally applicable, organizational strategies require systemic adaptation across different regulatory contexts. The study concludes that effective accident prevention necessitates a paradigm shift from behavioral compliance to systemic resilience, advocating for the integration of advanced engineering controls with proactive, system-wide psychosocial risk management.

## 1 Introduction

Coal remains a pivotal fuel for electricity generation in many parts of the world and continues to underpin energy systems that rely on thermal power plants. The coal logistics chain which includes international shipment, port handling, stockpiling, conveyor transfer, processing, and feeding into boilers constitutes a complex network of operations that exposes workers to a variety of occupational hazards along different stages of the supply chain. Workers involved in these logistics and handling operations may be exposed to risks such as moving machinery and conveyors, heavy lifting, vehicle–pedestrian interactions, airborne coal dust and explosion risks, falls from height, confined-space exposures, fire and explosion hazards, and ergonomic strain due to manual handling (1–4). Given this intricate hazard landscape, international safety standards and guidance recommend a systematic, integrated risk-management approach to identify, assess, control, and monitor risks in order to reduce workplace injuries and prevent serious or fatal incidents (5, 6).

For the purpose of this review, “coal logistics” is operationally defined as the post-extraction supply chain processes. This encompasses inland transportation (including rail, heavy-haul trucks, and mobile plant operations), bulk material handling via conveyor systems, stockpiling management, and maritime port operations (including ship loading and unloading) (3). By establishing this specific boundary, this study explicitly distinguishes the logistical hazard landscape from general upstream extraction safety issues, such as rock bursts or face blasting, which possess fundamentally different risk profiles (7).

Work-related injuries and fatalities remain a major global concern. According to recent estimates by the International Labour Organization (ILO), nearly three million workers die each year due to work-related accidents or diseases, and hundreds of millions more sustain non-fatal injuries globally (8). These figures underscore the heavy human, social, and economic burden of inadequate occupational safety and health, especially in high-risk industries. Industrial sectors involving extraction, heavy material handling, energy production, mining and logistics contribute disproportionately to fatal occupational injuries relative to their share of total employment, which highlights the high-risk nature of energy-sector operations (9).

Empirical research on coal-handling, mining, and related logistics operations further reinforces the significance of risk in these contexts. Several studies document recurring patterns of accidents resulting from maintenance activities near moving conveyors or heavy machinery, inadequate guarding of equipment, poor human–machine interaction design, and lack of hazard recognition or behavioral compliance (3, 10, 11). These findings point toward a need for comprehensive accident-prevention strategies that integrate engineering, administrative and human-factor controls (3, 12, 13).

Current occupational safety-management frameworks and international standards, such as ISO 45001:2018, provide structured approaches to manage occupational risks in high-hazard industries (14–16). These frameworks promote systematic hazard identification, formal risk assessment, implementation of control and mitigation measures, emergency preparedness, stakeholder and contractor management, compliance with regulatory and standards requirements, and continuous monitoring and improvement through Plan–Do–Check–Act cycles. Evidence from occupational safety research suggests that multi-component interventions, combining engineering safeguards, administrative procedures, training and behavioral interventions tend to yield stronger safety outcomes and more sustainable injury reduction compared with isolated single-factor measures, though the efficacy and generalizability of such interventions vary depending on context and operational conditions (17–19). However, it is critical to recognize that relying heavily on administrative procedures often provides only modest levels of safety performance. The persistence of poor procedures and inherent biases in accident investigations frequently hinder effective risk mitigation, demonstrating that procedural compliance alone is insufficient to prevent complex logistical accidents (20, 21).

Although research on coal-mine safety, machinery hazards, port-handling risks and human-factor issues is growing, existing studies are often fragmented and lack a unified perspective. Many focus on specific hazard types or isolated parts of the coal-handling chain, while others adopt narrow methodological approaches that limit the synthesis of broader insights. As a result, there is a notable gap in the literature for a comprehensive systematic review that consolidates global evidence to identify the critical elements of occupational accident risk management relevant to coal logistics operations. Addressing this gap is essential to establish an integrated understanding of risk-management requirements, especially for high-risk operations in coal logistics operations. Given these challenges, this systematic literature review aims to identify and synthesize the key elements of occupational accident risk management within the coal-logistics operations.

## 2 Methodology

This section outlines the methodology used to get publications pertaining to the occupational accidents risk management in coal logistics operations. The research methodology employed in this study was derived from the PRISMA 2020 framework. This framework encompasses the development of review questions, a systematic approach to conducting a bibliographic search (including identification, screening, and eligibility criteria), assessment of the quality of the selected studies, and the extraction and analysis of data. The study was conducted from 21 November 2025 till 13 December 2025.

### 2.1 Formulation of the research questions

According to Page et al. (22), the first step of a systematic review is to formulate an explicit statement of the research questions. The PICo method was used to develop research questions in this study. PICo is a tool that focuses on the population, interest, and context of a (usually quantitative) article (19). Based on these three main focuses of the PICo method, three main aspects were considered for this study, namely the coal logistics operations (population), occupational accidents (interest) and risk management (context). This current study is guided by the following research questions:

1. What are the predominant hazards and types of occupational accidents reported in coal logistics operations?
2. What risk management practices, strategies, or frameworks have been implemented to prevent or reduce occupational accidents in coal logistics operations?

### 2.2 Systematic searching strategies

**Figure 1:**
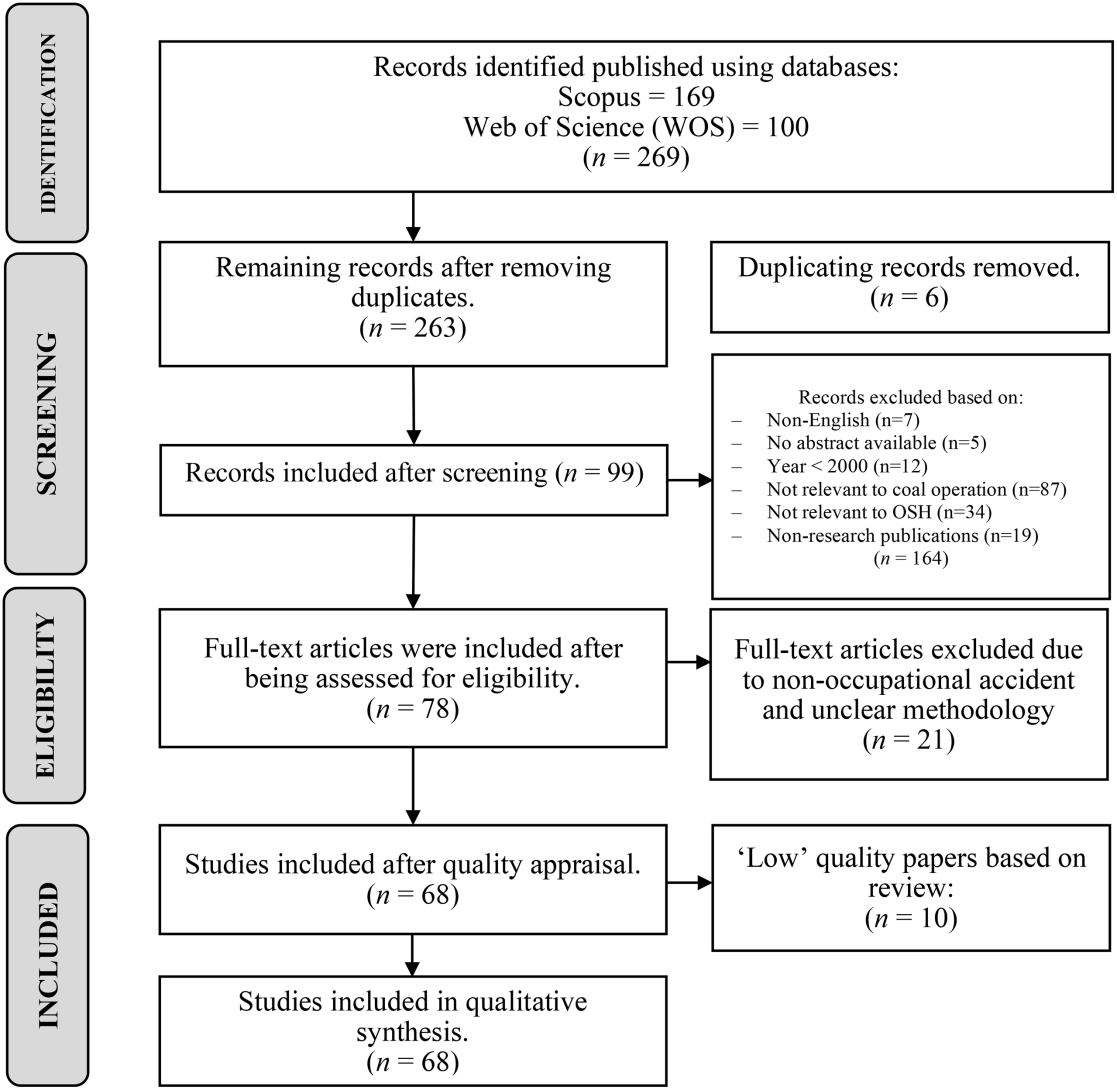
PRISMA 2020 statement flow diagram chart for systematic literature reviews adapted from Page et al. (22).

Page et al. (22) highlighted that the systematic search strategy consists of three main processes, which are: (1) identification of relevant publications required by the study (identification); (2) screening of acquired publications from electronic databases (screening); and (3) eligibility of screened publications (eligibility). Figure 1 illustrates the PRISMA 2020 Statement flow diagram chart for systematic literature reviews of this study.

#### 2.2.1 Identification and search strategy

The identification phase involved a systematic and comprehensive search strategy designed to retrieve high-impact literature relevant to the research questions defined by the PICo framework. To ensure robust coverage of the topic, the search strategy focused on three core conceptual clusters: (1) Coal Logistics Operations (Population), (2) Occupational Accidents (Interest), and (3) Risk Management (Context).

*Keyword Development:* The search terms were iteratively developed based on controlled vocabulary (e.g., MeSH terms) keywords from seminal studies and expert consultation (23). To minimize selection bias and capture all relevant synonyms lexical variations and related terminologies were included

- Cluster 1 (Coal Logistics): Keywords included ‘coal logistics’, ‘coal handling’, ‘coal supply chain’, ‘coal terminal’, ‘coal transportation’, ‘coal conveyor’, and ‘bulk material handling’.
- Cluster 2 (Accidents): Keywords included ‘occupational accident*’, ‘workplace accident*’, ‘industrial accident*’, ‘occupational injury’, ‘work-related injury’, ‘fatal accident*’, and ‘near miss’.
- Cluster 3 (Risk Management): Keywords included ‘risk management’, ‘risk assessment’, ‘hazard identification’, ‘safety management system’, ‘ISO 45001’, and ‘accident prevention’.

*Database Selection:* The search was conducted in Scopus and Web of Science (WoS). These databases were selected for their extensive multidisciplinary coverage, indexing high-impact journals in safety science, engineering, and environmental health, thus ensuring the retrieval of peer-reviewed and methodologically rigorous studies (24).

*Search Execution*: Advanced search techniques were employed using Boolean operators and truncation. The operator “OR” was used to combine synonyms within each cluster, while “AND” was used to link the three clusters (Logistics AND Accidents AND Risk Management). Truncation symbols (e.g., accident*) were utilized to capture plural forms and variations. The search was executed in November 2025, spanning titles, abstracts, and keywords. The specific search strings used for each database are detailed in Table 1. This initial search yielded a total of 269 records (Scopus = 169; WoS = 100).

**Table 1:** Search strings used in electronic databases.

Database	Search string
SCOPUS	(TITLE-ABS-KEY(“coal logistics” OR “coal handling” OR “coal supply chain” OR “coal terminal” OR “coal transportation” OR “coal conveyor*” OR “bulk material handling”)) AND (TITLE-ABS-KEY(“occupational accident*” OR “workplace accident*” OR “industrial accident*” OR “occupational injury” OR “work-related injury” OR “safety incident*” OR “fatal accident*” OR “near miss”)) AND (TITLE-ABS-KEY(“risk management” OR “risk assessment” OR “hazard identification” OR “safety management system” OR “ISO 45001” OR “accident prevention”))
Web of Science (WoS)	TOPIC: (“coal logistics” OR “coal handling” OR “coal supply chain” OR “coal terminal” OR “coal transportation” OR “coal conveyor*” OR “bulk material handling”) AND Refined by: TOPIC: (“occupational accident*” OR “workplace accident*” OR “industrial accident*” OR “occupational injury” OR “work-related injury” OR “safety incident*” OR “fatal accident*” OR “near miss”) AND TOPIC: (“risk management” OR “risk assessment” OR “hazard identification” OR “safety management system” OR “ISO 45001” OR “accident prevention”))

#### 2.2.2 Screening

The screening process constituted the second phase of the systematic search strategy, designed to filter the identified records based on their relevance to the specific scope of coal logistics safety. Initially, the 269 records retrieved from the electronic databases were exported to Mendeley Reference Manager (Mendeley Ltd., Elsevier Inc.) to identify and eliminate duplicates. A total of six duplicate records were removed, leaving 263 unique publications for the primary screening phase.

Subsequently, a two-stage screening procedure was conducted. In the first stage, the titles and abstracts of the remaining 263 articles were screened against the pre-defined inclusion and exclusion criteria detailed in Table 2. This process was critical to distinguish between general mining extraction research and the specific domain of logistics operations.

**Table 2:** The inclusion and exclusion criteria.

Category	Inclusion Criteria	Exclusion Criteria
Publication type	Indexed journal (articles, research, review) and conference paper	Chapters in book, book, editorial, mini review, news, report, short survey, note
Language	Publications written in English	Non-English publications
Availability	Full abstract available for screening	No abstract available
Publication timeline	Studies published 2000 and above	Studies published before 2000
Relevance to topic	Studies directly or indirectly related to: Coal logistics operations Occupational safety and health issues Occupational accident analysis	Studies unrelated to fire, focusing solely on: Natural disaster Not related to occupational safety
Study type	Empirical, experimental, numerical, simulation, modelling, or case-study research relevant to occupational accidents in coal logistics operations	Opinion papers, editorials, non-scientific articles, posters, or studies lacking methodological detail

To ensure the review’s rigor and focus, the following selection criteria were strictly applied:

1. Relevance to Logistics Operations: Studies were only included if they addressed hazards within the supply chain (transportation, handling, storage). Articles focusing exclusively on upstream extraction activities such as rock bursts, drilling safety, or face blasting were excluded as they represent a distinct risk profile unrelated to logistics.
2. Publication Type and Quality: To maintain the academic integrity of the review, inclusion was limited to peer-reviewed journal articles and conference proceedings. Non-peer-reviewed sources, including book chapters, editorials, news reports, and short surveys, were excluded.
3. Language and Timeline: Only English-language publications were selected to ensure accurate data extraction and synthesis. The timeline was restricted to studies published from the year 2000 onwards to capture relevant technological advancements and modern safety management practices.

Following this rigorous screening of titles and abstracts, 164 records were excluded for failing to meet the criteria (e.g., non-English, off-topic, or lacking an abstract). Consequently, 99 publications were retained and advanced to the eligibility phase for full-text assessment.

#### 2.2.3 Eligibility and full-text assessment

The third phase of the systematic search strategy involved the retrieval and critical examination of the full texts for the 99 publications retained from the screening stage. Unlike the preliminary screening, this phase focused on verifying the substantive relevance and methodological rigor of the studies against the specific operational definitions of ‘coal logistics’.

Each article was scrutinized to ensure it provided empirical or analytical data specifically related to the supply chain (transportation, handling, and storage) rather than general mining activities. A strict exclusion protocol was applied at this stage to remove studies that:

1. Scope Mismatch: Upon full reading, focused primarily on upstream extraction hazards (e.g., rock bursts, face ventilation) without distinct data on logistics operations.
2. Outcome Irrelevance: Discussed logistics equipment (e.g., conveyors) solely from an efficiency or mechanical maintenance perspective, without addressing occupational safety or accident risks.
3. Methodological Deficiency: Lacked clear research methods or empirical data, such as purely descriptive industry reports or theoretical opinion pieces without validatable evidence.

Based on this rigorous full-text assessment, 21 articles were excluded from the review. The primary reasons for exclusion included a lack of specific focus on occupational accidents and unclear methodological frameworks. Consequently, 78 articles satisfied all eligibility criteria and were advanced to the quality appraisal stage.

### 2.3 Quality appraisal

The quality appraisal process was designed to ensure the integrity and validity of the synthesized data. Given the methodological heterogeneity of the included studies, which comprise empirical research, simulations, and case studies, the Mixed Methods Appraisal Tool (MMAT) version 2018 was employed (25). The MMAT is specifically designed for systematic reviews that include qualitative, quantitative, and mixed-methods studies.

Two independent reviewers critically assessed each article against the specific MMAT criteria for its respective study design. To enhance transparency, an explicit scoring threshold was established: studies meeting 80 to 100% of the MMAT criteria were categorized as ‘High’ quality; those meeting 60 to 79% were categorized as ‘Medium’ quality; and studies scoring below 60% were categorized as ‘Low’ quality. Any discrepancies between the reviewers were resolved through discussion until a consensus was reached. Ultimately, 10 articles categorized as ‘Low’ quality, primarily due to insufficient methodological detail and lack of clear exposure-outcome definitions, were excluded. Consequently, 68 peer-reviewed articles (55 High, 13 Medium) were retained for the final qualitative synthesis.

## 3 Results

### 3.1 The general findings and background of the selected articles

**Figure 2:**
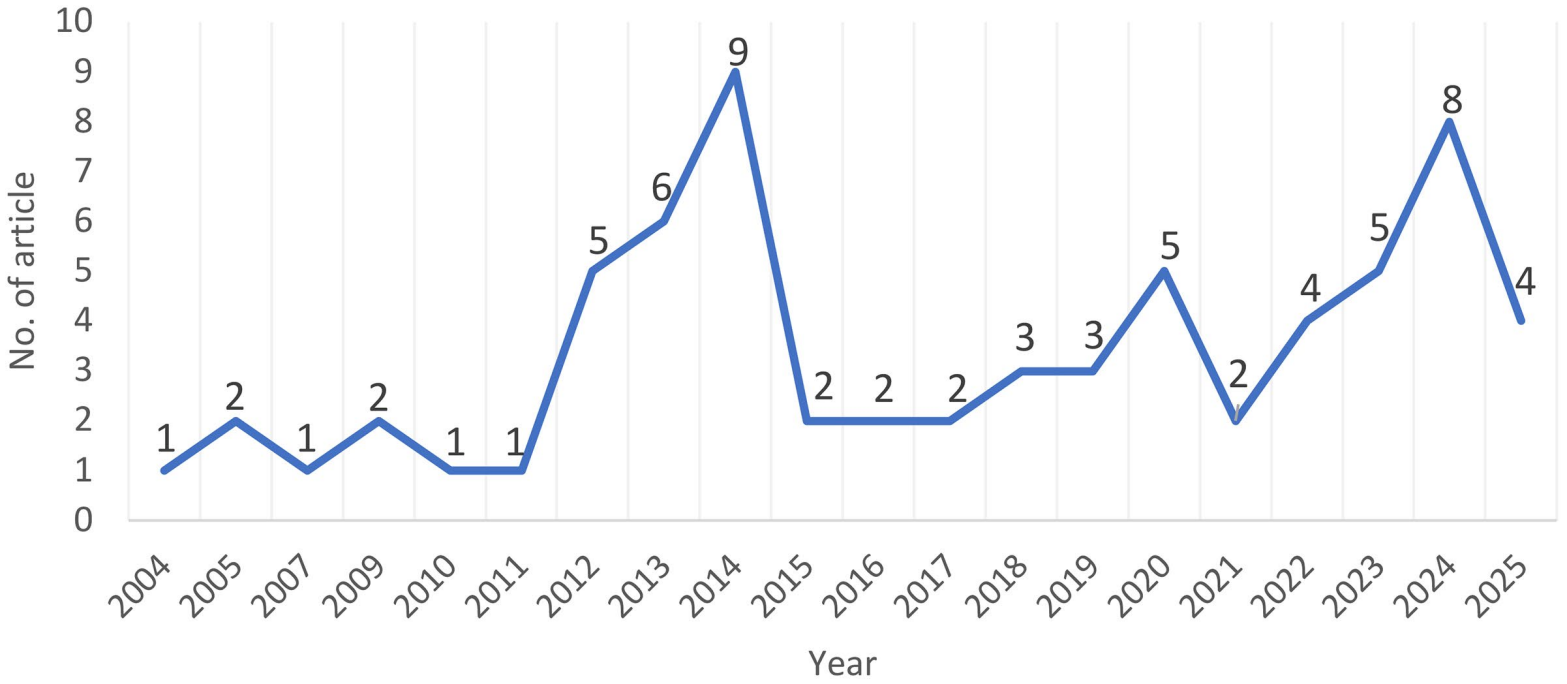
The number of reviewed articles selected by year published.

The temporal distribution of the reviewed articles provides an overview of the publication trends related to occupational hazards and accident risks in coal logistics operations. A total of 68 articles published between 2004 and 2025 were included in this systematic literature review. The annual distribution of these articles is presented in Figure 2.

As shown in Figure 2, early publications in this research area were relatively limited. Between 2004 and 2011, the number of articles published per year remained low, ranging from one to two articles annually, indicating sporadic scholarly attention during this period. A gradual increase in publication output becomes evident from 2012 onwards, with five articles published in 2012 and six articles in 2013.

The number of publications reached an initial peak in 2014, with nine articles, representing the highest annual output within the reviewed time frame. Following this peak, the publication volume declined and stabilized at a lower level between 2015 and 2017, with two articles published each year. From 2018 to 2021, the number of articles fluctuated moderately, ranging between two and five publications per year, indicating a sustained but variable level of research activity.

As further illustrated in Figure 2, a renewed increase in publication output is observed from 2022 onwards. Specifically, four articles were published in 2022, followed by five articles in 2023 and eight articles in 2024, marking a second notable rise in annual publication frequency. In 2025, up to the time of data collection, four articles were identified and included in the review.

**Figure 3:**
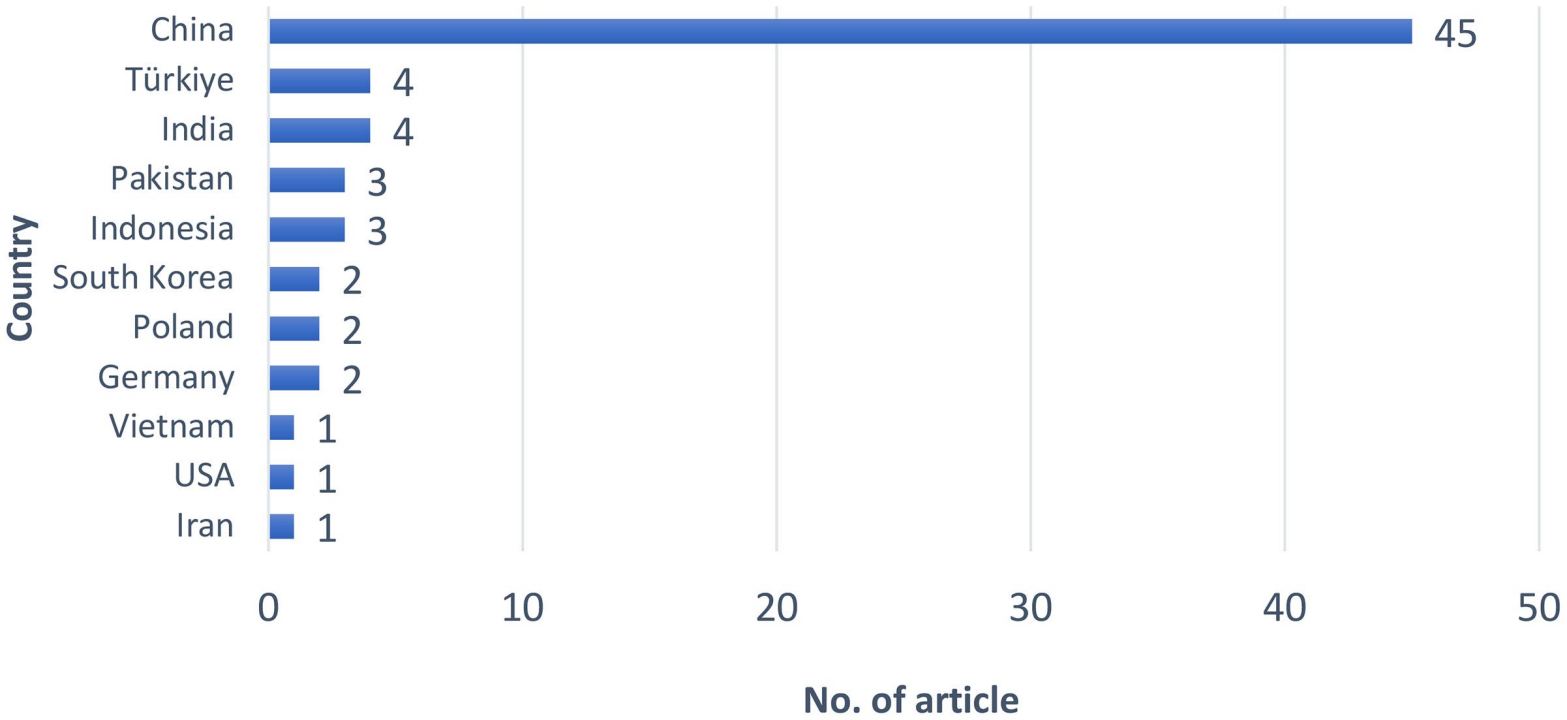
The number of reviewed articles selected by country.

The geographical distribution of the reviewed articles provides insight into the countries contributing to research on occupational hazards and accident risks in coal logistics operations. A total of 68 articles were included in this systematic literature review, with their country of origin summarized in Figure 3.

As illustrated in Figure 3, the reviewed literature is heavily concentrated in a small number of countries. China accounts for the largest share of publications, contributing 45 articles, which represents a substantial majority of the total studies reviewed. This dominant contribution indicates a strong research focus on coal logistics–related safety issues within the Chinese context.

Beyond China, a moderate level of research output is observed in several countries. India and Türkiye each contributed four articles, followed by Indonesia and Pakistan with three articles each. A smaller number of publications originated from Germany, Poland, and South Korea, with two articles reported for each country.

In contrast, limited contributions are identified from Iran, the United States, and Vietnam, each represented by one article in the reviewed dataset. These findings highlight a relatively uneven geographical distribution of research outputs, with a small number of countries accounting for the majority of published studies.

### 3.2 Thematic analysis of hazards and occupational accidents in coal logistics operations

**Figure 4:**
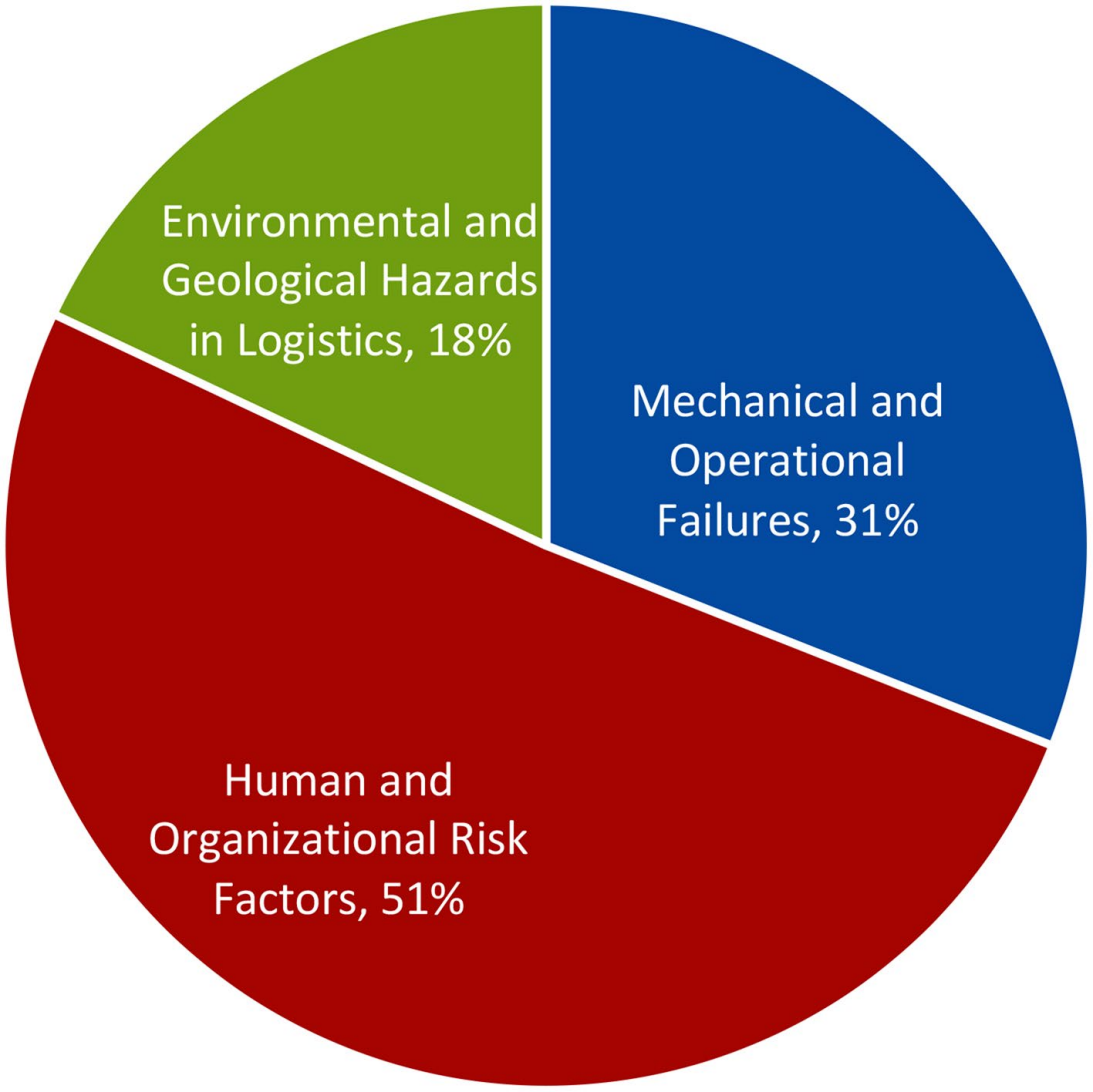
Themes of occupational hazards.

From all the 68 articles, a total of three themes of occupational hazards were extracted: (a) Mechanical and Operational Failures, (b) Human and Organizational Risk Factors, and (c) Environmental and Geological Hazards in Logistics. The distribution of the 68 selected articles into these three themes is illustrates in Figure 4. The themes were derived based on the analysis conducted across all the 68 selected articles. The most studied occupational hazards theme in coal logistics operations was ‘Human and Organizational Risk Factors’ with 51% (35 articles), followed by ‘Mechanical and Operational Failures’ with 31% (21 articles), and ‘Environmental and Geological Hazards in Logistics’ with 18% (12 articles).

#### 3.2.1 Human and organizational risk factors

Human and organizational risk factors constitute a major category of occupational hazards in coal logistics operations, as consistently reported across the reviewed studies. The results indicate that accidents within coal logistics systems are frequently underpinned by human-related failures, often interacting with organizational conditions such as work design, supervision structures, and psychosocial work environments. These factors are commonly identified as root or initiating causes of logistical accidents rather than as isolated contributory elements.

Human factors is an inherently broad term that requires specific breakdown to be meaningful in the context of coal logistics. As (26) argues, listing “human error” as a root cause is far too vague to be useful because different types of errors require entirely different preventive actions. Following established human error classification models (27), human factors must be analyzed beyond surface-level mistakes. Therefore, rather than classifying human-related hazards under a generic umbrella, this review granulates these factors into the following three critical sub-categories:

- Cognitive workload and decision-making

The reviewed literature provides strong quantitative evidence indicating the predominance of human error in coal-related accidents. In studies analyzing coal mine accidents in China, human factors account for approximately 71.6% of all accidents, positioning them as the most significant causal category when compared with equipment-related or environmental factors (28). Similarly, in the Indian coal mining context, human error is reported as the most influential causal risk factor, with a risk weight of 0.26, exceeding those associated with environmental hazards and equipment failures (29).

Logistics operators, particularly those managing complex dispatch systems or heavy mobile equipment, frequently suffer from cognitive overload due to high task demands and time pressures. This overload impairs situational awareness and delays reaction times during critical incidents. Within this category, unsafe acts are identified as the primary manifestation of human-related hazards, encompassing both unintentional errors and intentional violations of established rules (30). Empirical analyses indicate that illegal operation is the most frequently observed unsafe act, accounting for 71.9% of total unsafe acts identified in specific accident datasets (31). However, to effectively prevent accidents, safety management must address the underlying causes of these organizational violations. The International Association of Oil and Gas (32) emphasizes a critical human performance principle: context drives behavior. Research into fatal industrial accidents reveals that most safety infringements are situational rather than intentional (33). Frequently, employees commit violations not for personal gain, but because they believe their actions will benefit the organization by maintaining operational efficiency under extreme production pressure (34).

- Fatigue and psychosocial stress

Beyond observable unsafe acts, the reviewed literature also identifies the unsafe state of workers as a critical human-related hazard condition (30). These states are characterized by physical fatigue, mental strain, reduced alertness, and impaired cognitive functioning. Prolonged working hours and shift work inherent in continuous coal logistics operations, such as 24-h port operations and long-haul transport, heavily contribute to physical and mental fatigue (30).

High workload, encompassing both long working hours and high task demands, demonstrates a statistically significant positive relationship with the emergence of unsafe worker states (35). This fatigue correlates directly with diminished concentration and increased accident susceptibility during logistics operations (35). Furthermore, job dissatisfaction is reported as a contextual organizational factor that intensifies the relationship between workload and unsafe states (35).

- Ergonomics and physical strain

Workers engaged in material handling and equipment operation face significant ergonomic hazards. The reviewed literature documents whole-body vibration (WBV) as a recurring human-related hazard, particularly affecting haulage operators engaged in coal logistics operations (36). Chronic exposure to WBV, especially along the vertical axis, is consistently associated with the development of musculoskeletal disorders (MSDs) (36).

Reported symptoms commonly include pain in the lower back, elbows, and knees. In addition to physical exposure, several studies identify psychosocial work factors, such as high job demands and limited control at work, as conditions associated with an increased prevalence of WBV-related MSDs (37). This positions WBV not only as a physical hazard but also as a human-organizational risk factor that reflects the combined effects of task demands, work organization, and prolonged equipment operation.

#### 3.2.2 Mechanical and operational failures

Mechanical and operational failures emerge as a distinct category of occupational hazards within coal logistics operations, as evidenced by the recurring patterns reported across the reviewed studies. This category encompasses hazards arising from the malfunction, degradation, or extreme operational loading of mechanical systems that support coal transportation, handling, and transfer processes. The findings indicate that such failures are prevalent across different logistics contexts, including in-mine haulage systems, bulk material handling infrastructure, and port-based coal logistics operations.

- Conveyor belt system failures

Among mechanical hazards, failures associated with conveyor belt systems are frequently reported in the literature. Conveyor belts, as core components of bulk coal handling operations, are exposed to continuous operation under harsh environmental conditions. One of the most commonly identified failure modes is belt deviation or mistracking, which occurs when the belt moves laterally away from its intended alignment (38). Studies consistently classify belt deviation as a significant mechanical hazard due to its association with material spillage, abnormal belt-structure interaction, and unintended contact with rotating components.

The reviewed studies further report that the detection of belt deviation during operation is constrained by environmental conditions typical of coal logistics environments, including high dust concentration, limited visibility, and variable lighting (38). These conditions are repeatedly cited as contributing factors to delayed identification of conveyor system failures. As a result, belt deviation is frequently documented not as an isolated mechanical fault but as an operational failure that develops progressively during continuous system use (38).

- Heavy haulage equipment and dynamic load failures

Mechanical and operational failures are also prominently reported in relation to heavy haulage equipment used in coal logistics, particularly in high-capacity handling systems such as gantry hoists, ship unloaders, and large-scale transport machinery (39). The literature identifies extreme dynamic internal forces, commonly referred to as shock loads, as a recurring source of mechanical stress within these systems. Such forces are most pronounced during emergency or rapid braking events, which subject drivetrain components to sudden and substantial load variations (39).

Across the reviewed studies, drivetrain-related components especially roller bearings within hoist gearings are frequently reported as failure-prone elements under high dynamic loading conditions. These failures are characterized by accelerated wear, fatigue damage, and, in some cases, abrupt component breakdown. The accumulation of operational stress under repeated braking cycles is consistently highlighted as a key mechanical hazard intrinsic to the operation of heavy coal logistics equipment.

- Operational stress in mobile coal transportation systems

In addition to stationary and semi-stationary equipment, mechanical and operational failures are also documented in mobile coal transportation systems, particularly in open-pit mining environments. Studies report that coal haulage trucks operate under highly variable conditions involving fluctuating speeds, gradients, payloads, and road surface quality (38). These operational characteristics contribute to mechanical stress on braking systems, drivetrains, and suspension components.

The reviewed literature identifies overspeeding and speed variability as operational conditions associated with increased mechanical loading and heightened failure likelihood. Such conditions are not described as behavioral issues but are reported as operational states that place sustained stress on vehicle systems. These stress-induced conditions are frequently linked to mechanical degradation over time, thereby forming part of the broader category of mechanical and operational hazards.

- Monitoring and detection-related operational failures

Several studies included in the review also categorize limitations in real-time monitoring and detection as an operational failure contributing to mechanical hazard manifestation (40). This includes delayed or inaccurate identification of equipment position, movement, or operational state during coal logistics activities. For example, challenges in accurately detecting conveyor belt edges or tracking coal transport vehicles under adverse environmental conditions are reported as recurrent operational constraints.

Such monitoring limitations are consistently framed in the literature as intrinsic operational challenges associated with coal logistics environments rather than as management deficiencies. They are documented as contributing factors that allow mechanical faults or abnormal operating conditions to persist undetected, thereby increasing exposure to mechanical hazards.

#### 3.2.3 Environmental and geological hazards in logistics

Environmental and geological hazards represent a distinct category of occupational hazards within coal logistics operations, as reported across the reviewed studies. This category encompasses risks arising from the physical and natural environment in which logistics activities are conducted, including underground geological conditions, surface environmental factors, and material storage environments (7, 41). The results indicate that these hazards frequently interact with logistics systems, directly influencing the safety of transportation routes, material handling processes, and operational continuity.

- Ground conditions and geological complexity in underground logistics

The reviewed literature consistently identifies ground conditions and geological complexity as inherent hazards in underground coal logistics operations (29, 41). Deep mining environments are characterized by complex geological structures, including variable strata strength, fault zones, and stress redistribution, all of which complicate both production and transportation activities. These conditions directly affect the stability and integrity of underground haulage roadways, creating persistent exposure to geological hazards during logistics operations.

Accidents associated with ground movement, such as roof falls, roadway collapses, and slope instability, are frequently classified as a major accident category in coal mining accident statistics. In the Indian coal mining context, ground movement related accidents carry a risk weight of 0.232, positioning them among the most significant accident classes reported (29). These incidents directly disrupt underground logistics routes and are consistently associated with severe consequences due to sudden structural failure in confined environments.

The reviewed studies report that such geological hazards are not episodic but intrinsic to underground logistics systems, with risks varying spatially and temporally as excavation progresses. As a result, ground condition–related hazards are repeatedly documented as a persistent environmental risk affecting coal transportation activities below ground.

- Weather-related hazards in surface coal logistics

For surface-based coal logistics operations, adverse weather conditions are consistently reported as critical environmental hazards. The reviewed studies identify meteorological factors such as heavy rainfall, snowfall, fog, and strong winds as conditions that directly influence the safety of haulage operations (38). These weather events are reported to degrade road surface conditions, reduce tire-road adhesion, impair visibility, and affect vehicle stability.

Accident reports and observational studies included in the review document increased incident occurrence during periods of adverse weather, particularly in open-pit mining and surface transportation corridors (38). Heavy rainfall is frequently associated with slippery road surfaces and increased braking distances, while fog and dust storms significantly reduce driver visibility. Strong winds are reported as destabilizing factors for high-sided vehicles and material handling equipment. Collectively, these weather-related hazards are identified as environmental conditions that heighten exposure to accidents during coal logistics operations (42–44).

- Stockpiling and material handling environmental hazards

The storage and preparation stages of coal logistics introduce a separate set of environmental hazards related to stockpiling and material handling. Among these, the self-ignition or spontaneous combustion of coal stockpiles is repeatedly reported as a severe safety, economic, and environmental hazard within logistics chains (45). The reviewed literature identifies spontaneous combustion incidents as a significant risk in both mine-site and port-based coal logistics facilities.

The studies consistently report that the physical characteristics of coal stockpiles are primary intrinsic factors influencing self-ignition risk (45). Key characteristics include pile height, bed porosity, and side slope geometry. High bed porosity and steep side slopes are repeatedly associated with increased oxygen ingress and reduced heat dissipation, creating favorable conditions for heat accumulation and spontaneous combustion (45).

In addition to intrinsic pile characteristics, meteorological conditions are identified as extrinsic environmental factors affecting self-ignition risk (45, 46). Ambient temperature, wind velocity, and humidity are frequently reported as influencing the rate of heat generation and dissipation within coal piles. Higher ambient temperatures and increased airflow are commonly associated with accelerated self-heating processes, while variations in humidity affect oxidation rates. These environmental conditions are consistently documented as contributing to the temporal variability of spontaneous combustion incidents in coal logistics operations.

### 3.3 Thematic analysis of risk management practices or strategies to prevent occupational accidents in coal logistics operations

From systematic review of all 68 articles, a total of four themes of risk management practices or strategies to prevent occupational accidents in coal logistics operations were extracted: (a) Systemic & Organizational Management Frameworks, (b) Human Factors and Behavioral Control, (c) Technical and Operational Mitigation, and (d) Material and Environmental Hazard Control. The distribution of the 68 selected articles into these four themes and its sub-themes is shown in Table 3. The percentage calculation for each theme and sub-theme is based on 68 articles. The percentage indicates the frequency with which the theme was discussed substantively across all 68 articles. Because one article can discuss several themes/ subthemes (for example, one article discusses belt deviation technology and employee training), the total percentage of individual themes can exceed 100%.

**Table 3:** Themes and sub-themes for risk management practices or strategies to prevent occupational accidents.

Theme and sub-theme	Prevalence (n/N = 68)	Percentage (%)
**T1: Systemic and organizational management frameworks**	**51**	**75%**
T1.1: Formal safety management systems (SMS)	42	61.8%
T1.2: Risk assessment and dynamic monitoring	31	45.6%
T1.3: Data-driven and AI management	25	36.8%
T1.4: Eliminating Backward Capacity and Increasing Investment	14	20.6%
**T2: Human factors and behavioral control**	**48**	**70.6%**
T2.1: Training, competence, and access control	36	52.9%
T2.2: Behavioral and disciplinary mechanisms	32	47.1%
T2.3: Psychosocial and health management	28	41.2%
**T3: Technical and operational mitigation**	**20**	**29.4%**
T3.1: Intelligent equipment and automation	17	25.0%
T3.2: Advanced monitoring and detection	14	20.6%
T3.3: Equipment redesign and performance	5	7.4%
**T4: Material and environmental hazard control**	**11**	**16.2%**
T4.1: Stockpile and spontaneous combustion control	5	7.4%
T4.2: Geological hazard mitigation	5	7.4%

#### 3.3.1 Systemic and organizational management frameworks

This theme encompasses formalized strategies and top-down structures implemented to manage safety proactively across coal logistics operations. Systemic strategies focus on integrating safety into organizational processes using formalized, data-driven methods (47). There were four sub-themes under the systemic and organizational management frameworks namely: Formal Safety Management Systems (SMS) (*n* = 42, 61.8%), Risk Assessment and Dynamic Monitoring (*n* = 31, 45.6%), Data-Driven and AI Management (*n* = 25, 36.8%), Eliminating Backward Capacity and Increasing Investment (*n* = 14, 20.6%).

For formal Safety Management Systems (SMS), mandatory implementation and standardization of systems such as the Occupational Safety and Health Management System (OHSMS) is crucial for systematic risk governance (48, 49). Coal enterprises must establish a comprehensive management system where responsibilities are clearly implemented and reinforced (28). Furthermore, a dedicated safety management leadership team must be established, headed by the mine director, to organize and implement occupational safety and health management (48).

In term of Risk Assessment and Dynamic Monitoring, proactive risk control is achieved through comprehensive risk management frameworks, which transition safety management from passive/reactive to active and dynamic (50). This includes conducting risk identification through systematic methods like checklists, hazard evaluation methods, or system safety analysis (48). Once risks are identified, they are classified into grades (e.g., A, B, C) to prioritize treatment (48). A critical practice involves using network-based application software, such as the B/S mode system, for real-time dynamic monitoring of risk status (48, 50). This software supports risk early warning (e.g., scrolling information, flashing menus) and automatically issues control commands and timely notification to relevant managers, often via mobile phones, to optimize control measures.

For Data-Driven and AI Management, advanced analytical methods are increasingly applied to enhance risk prediction and logistical planning. This includes using data mining technology in combination with machine learning (ML) models like Rough Set-Wavelet Neural Network (RS-WNN) to predict coal mine production logistics system security states with high accuracy (51). Furthermore, ML algorithms like Random Forest (RF) are used to accurately predict open-pit mining truck speeds, aiding in scheduling optimization and the early prediction of hazardous situations like speeding (38). For complex logistics hubs like coal ports, AI techniques such as Constraint Programming are used to solve complicated operational scheduling problems, ensuring safety inventories are maintained and mitigating collision risks for moving equipment (52).

Pertaining to Eliminating Backward Capacity and Increasing Investment, a fundamental measure is the resolute elimination of backward production capacity and facilities, such as coal mines with low productivity or those using prohibited methods (50). Enterprises must increase safety input, dedicating funds to equipment renovation and technology upgrades to reduce accident frequency directly and effectively (50, 53). Scientific research, safety supervision, and investment are identified as key components for preventing major accidents (44).

#### 3.3.2 Human factors and behavioral control

Interventions directly targeting employee behavior and psychological states are prioritized, consistent with the identification of human factors as the primary cause of accidents (28, 54). Given that unsafe behavior is the predominant reason for a large percentage of safety accidents, risk management heavily relies on controlling and influencing workers’ actions and psychological states (52). Human factors and behavioral control consists of three sub-themes namely: Training, Competence and Access Control (*n* = 36, 52.9%), Behavioral and Disciplinary Mechanisms (*n* = 32, 47.1%), Psychosocial and Health Management (*n* = 28, 41.2%).

For Training, Competence and Access Control, strengthening safety education and training is paramount, specifically for new employees (clarifying safety laws and systems) and experienced employees (enhancing awareness to ensure legal, lawful, and reasonable work) (28, 50). This also involves enhancing the safety quality of managers to ensure they strictly implement safety systems and conduct thorough hazard investigations (28). Strict personnel access systems should be established, especially for underground work, requiring technical qualifications and specific experience (50).

Regarding to Behavioral and Disciplinary Mechanisms, management aims to restrain unsafe acts (such as the “three disobeying”: illegal command, illegal operation, and violation of labor discipline) through strict reward and punishment measures (positive incentives and reverse constraints) (28, 54). Supervision must be strengthened to detect and correct illegal operations in a timely manner (48, 50). Furthermore, establishing a system of returning and visiting workers who have performed unsafe behaviors is suggested to avoid recurrence (30).

In term of Psychosocial and Health Management, interventions address the internal state of miners, recognizing that high workload (time and demand) positively impacts the miner’s unsafe state (35). Strategies include monitoring the physiological and mental health of miners (e.g., fatigue, pressure, abnormal state) to identify risks promptly (28, 35). Improving job satisfaction is a crucial moderating strategy, as a higher job satisfaction level significantly reduces the positive impact of workload on a miner’s unsafe state (35). Management must provide sufficient rest time and recreational activities to combat physical and mental fatigue (35).

#### 3.3.3 Technical and operational mitigation

This theme focuses on engineering controls, mechanization, and the integration of specialized monitoring technology, particularly relevant to core logistics assets like conveyors and haulage equipment. Technological advancements are focused on replacing human presence in hazardous logistics tasks and optimizing system efficiency and safety through monitoring. Among sub-themes for Technical and Operational Mitigation are: Intelligent Equipment and Automation (*n* = 17, 25.0%), Advanced Monitoring and Detection (*n* = 14, 20.6%), Equipment Redesign and Performance (*n* = 5, 7.4%).

For Intelligent Equipment and Automation, Coal mining enterprises must accelerate intelligent construction to achieve the goal of “replacing people with mechanization and reducing people with intelligence” (28). Despite this ambitious goal, the feasibility of completely replacing human operators remains highly debatable. Recent perspectives argue that advanced technology and artificial intelligence are more likely to complement, rather than entirely replace, human workers in complex environments (55). Human oversight remains essential to manage unpredictable logistical disruptions that automated systems cannot yet resolve. This includes upgrading equipment to achieve “0 failure” operational records through rigorous inspection and maintenance (28). The trend involves transitioning logistics toward highly intelligent and artificial intelligence-based systems (56).

Related to Advanced Monitoring and Detection, implementation of safety monitoring and inspection equipment, such as acoustic and electrical systems and advanced monitoring and early warning equipment (28). Specific to logistics, advanced methods are used for real-time belt deviation detection based on deep learning models (RBDNet) using depth edge features and gradient constraints, which offers high accuracy and speed even in dusty environments (38). Furthermore, technologies like UAVs and YOLOv9 are used for detecting and monitoring coal trucks to improve surveillance against traffic violations and congestion (40).

In term of Equipment Redesign and Performance, for safety-critical logistics equipment, redesign strategies focus on reducing shock loads. This involves implementing “intelligent braking” with synchronized and balanced action of all participating brakes (service brake and safety brake) to minimize high dynamic internal forces on drive trains (e.g., hoist gearings and roller bearings) during emergency stops (39). Maintaining high component reliability is essential for reducing longwall system downtime caused by maintenance logistics delays (57).

#### 3.3.4 Material and environmental hazard control

This category addresses the unique material and geological threats inherent to the coal environment that impact logistical safety. Material and Environmental Hazard Control consist of two sub-themes namely: Stockpile and Spontaneous Combustion Control (*n* = 5, 7.4%), Geological Hazard Mitigation (*n* = 5, 7.4%).

Pertaining to Stockpile and Spontaneous Combustion Control, evaluation systems, often using Fuzzy AHP approaches, are implemented for comprehensive, systematic evaluation of self-ignition risks in coal stockpiles (45). Countermeasures derived from these analyses include reducing pile side slope, compaction, and implementing wind screens to manage oxygen availability and heat release (45).

Regarding to Geological Hazard Mitigation, to prevent disasters that compromise mine structure and transport routes, prevention requires strengthening geological exploration to achieve “zero error in disaster detection” before mining begins (28). Quantitative data mining methods like Statistical Process Control (SPC) and Logistic Regression are applied to accurately predict critical sensitivity values for major events like coal and gas outbursts (58).

## 4 Discussion

### 4.1 The pattern of occupational accidents risk management in coal logistics operations

The aim of this research was to conduct a systematic literature review to identify and synthesize the key elements of occupational accident risk management within coal logistics operations. Through a temporal distribution analysis, this study examines the patterns of occupational accident risk management published between 2004 and 2025. The findings demonstrate a clear upward trend in publications, particularly after 2018, whereas the limited number of studies prior to 2010 suggests that early research primarily focused on core mining extraction rather than the logistics supply chain. This recent increase reflects a growing recognition that coal logistics, which includes transportation, handling, and storage, pose significant occupational risks that require systemic management. This trend aligns with a broader shift in occupational safety and health (OSH) research from reactive accident investigations toward proactive and system based risk management approaches, supported by the adoption of international standards such as ISO 45001 (59–61).

A critical observation from the demographic analysis is the significant preponderance of research originating from China, which accounts for approximately 66% of the reviewed articles. While this dominance mirrors China’s status as the world’s largest coal producer and consumer, it raises important questions regarding the global generalizability of the identified risk management practices. The surge in Chinese literature correlates strongly with the nation’s aggressive state led interventions over the last decade, specifically emphasizing the elimination of backward production capacity and increasing safety investment as primary management strategies (28). Furthermore, the strategic push towards “intelligent construction” to replace human labor with mechanization reflects a specific regulatory ecosystem characterized by centralized governance and high capital investment in state-owned enterprises (28, 56).

#### 4.1.1 Geographic disparities and generalizability

The literature reveals a heavy geographical concentration in China alongside contributions from other developing nations such as India, Indonesia, Pakistan, and Türkiye. These countries are often characterized by a high dependency on coal and emerging safety management systems. This geographical skew introduces variations in safety standards because risk management strategies that rely heavily on centralized, top-down disciplinary mechanisms may differ significantly from the unionized, compliance driven frameworks observed in developed nations like Australia or the United States (28).

Furthermore, differences in national work cultures, such as the high-power distance prevalent in some Asian contexts compared to the flatter organizational hierarchies found in Western industries, strongly influence safety outcomes (62, 63). These cultural factors determine whether workers feel empowered to report near misses or have the psychological safety to refuse unsafe commands. The relatively low number of studies from developed countries, such as the USA and Germany, may reflect more mature regulatory frameworks and a gradual transition toward alternative energy sources. Therefore, while the technical findings of this review are robust, the organizational and regulatory safety interventions must be carefully adapted to different national and cultural contexts to ensure their effectiveness (28).

#### 4.1.2 The impact of digitalization and industry 4.0

The notable surge in publication output observed from 2022 onwards is strongly linked to the advent of Industry 4.0 and digitalization within the coal sector. Particularly over the past 3 to 5 years, recent literature emphasizes a paradigm shift from manual monitoring to automated, data-driven safety management (64). The integration of the Internet of Things (IoT), artificial intelligence (AI), and deep learning models has revolutionized logistics safety. Beyond mere operational efficiency, these recent technological developments fundamentally support occupational safety by removing human workers from the line of fire. For example, technologies such as YOLOv9 for truck surveillance (40) and RBDNet for real time conveyor belt deviation detection (38) allow for predictive maintenance and the complete isolation of personnel from high-risk logistical zones. This technological evolution marks a critical transition toward autonomous supply chains and intelligent logistics systems (38, 40, 56, 64).

Despite this geographical and technological evolution, the technical validity of many findings remains robust across borders. Occupational hazards such as conveyor belt deviations (38) and spontaneous combustion in stockpiles (45) are governed by physical laws rather than geopolitical boundaries. Consequently, technical mitigation strategies like deep learning-based detection and real time sensors are universally applicable regardless of the operational location. In contrast, the organizational strategies identified, particularly those relying on strict behavioral compliance and punishment mechanisms, may require adaptation when applied outside the specific industrial contexts where they were developed.

### 4.2 Hazards and occupational accidents in coal logistics operations

The thematic analysis reveals that occupational accident risks in coal logistics operations are predominantly driven by human and organizational risk factors, followed by mechanical and operational failures, and environmental and geological hazard.

The strong emphasis on unsafe acts, particularly violations such as illegal operation and illegal command, indicates that compliance-based safety controls alone may be insufficient in coal logistics environments. The recurring presence of violations across different national contexts suggests systemic issues related to production pressure, supervision quality, and safety culture rather than individual worker negligence. Previous studies have similarly shown that violations are often normalized behaviors shaped by organizational expectations and operational constraints, especially in logistics-intensive and time-sensitive operations (62). Furthermore, the identification of unsafe worker states linked to high workload, fatigue, and job dissatisfaction highlights the importance of psychosocial risk factors, reinforcing calls for integrated OSH management approaches that address both physical and mental demands of work (63).

Mechanical and operational failures constitute the second most prominent hazard theme, reflecting the heavy reliance of coal logistics operations on continuous, high-capacity mechanical systems. The recurring failures of conveyor belts, haulage equipment, and braking systems demonstrate that these hazards are not merely technical in nature but are closely linked to operational stress, environmental conditions, and monitoring limitations. Similar findings in safety engineering literature indicate that prolonged operation under dynamic loads and harsh environments accelerates equipment degradation and increases accident likelihood when early warning systems are inadequate (65, 66). This suggests a need to move beyond reactive maintenance strategies towards predictive and condition-based monitoring in coal logistics systems.

Environmental and geological hazards, although less frequently studied, represent high-severity risks due to their inherent and often uncontrollable nature. Ground instability in underground logistics, adverse weather conditions in surface transport, and spontaneous combustion in coal stockpiles are consistently reported as hazards that interact with both human and mechanical systems. These findings support previous research indicating that environmental conditions often act as risk multipliers, exacerbating existing human and technical vulnerabilities rather than functioning as independent accident causes (7, 42). Consequently, effective risk management in coal logistics requires adaptive controls that account for environmental variability and dynamic operating conditions.

Overall, the discussion highlights that occupational accident risks in coal logistics operations are systemic and multi-dimensional. The interaction between human behavior, mechanical systems, and environmental conditions underscores the necessity for holistic risk management frameworks that integrate technical controls, organizational interventions, and environmental risk monitoring. This reinforces the relevance of system-based safety models and integrated OSH management systems in addressing the complex risk profile of coal logistics operations.

### 4.3 Risk management practices to prevent occupational accidents in coal logistics operations

After conducting an extensive analysis of specific articles using the PRISMA 2020, the findings indicate that risk management practices in coal logistics operations are predominantly concentrated on systemic and organizational management frameworks, followed closely by human factors and behavioral control. The strong emphasis on formal safety management systems (SMS), risk assessment, and dynamic monitoring suggests that coal logistics safety is increasingly managed through structured, top-down governance mechanisms rather than isolated technical interventions. This aligns with the broader evolution of occupational safety and health (OSH) management, where organizational systems and leadership commitment are recognized as foundational elements for effective accident prevention in high-risk industries (60, 61).

The high prevalence of human factors and behavioral control strategies further reflects the recognition that unsafe behavior remains a primary driver of occupational accidents in coal logistics operations. The extensive focus on training, competence assurance, and disciplinary mechanisms indicates that organizations prioritize direct control of worker behavior to mitigate accident risks. However, the inclusion of psychosocial and health management strategies highlights an important shift from purely compliance-based controls toward addressing underlying contributors to unsafe states, such as workload, fatigue, and job dissatisfaction. This supports existing evidence that behavioral safety interventions are most effective when integrated with organizational and psychosocial risk management approaches (59, 63).

Technical and operational mitigation measures, although less frequently reported, play a critical supporting role by reducing reliance on human intervention in hazardous logistics tasks. The adoption of intelligent equipment, automation, and advanced monitoring technologies reflects a preventive strategy consistent with the hierarchy of controls, where engineering solutions are preferred over administrative measures. The relatively lower emphasis on equipment redesign and performance improvement suggests that technological risk management in coal logistics is still evolving and may be constrained by high capital costs and legacy infrastructure (65, 66).

Material and environmental hazard control strategies are the least represented theme, despite the potentially severe consequences associated with geological instability and spontaneous combustion of coal stockpiles. This imbalance indicates that environmental risks in coal logistics are often treated as inherent or residual hazards rather than as risks that can be systematically managed through integrated control strategies. Previous studies have similarly noted that environmental hazards tend to receive less managerial attention unless they directly trigger major accidents, underscoring the need for stronger integration of environmental risk monitoring within logistics safety management sys (1–4).

Overall, the thematic distribution of risk management practices suggests that coal logistics safety is primarily governed through organizational and behavioral controls, supported by selective technological interventions. While this approach reflects current best practices in OSH management, the findings highlight the need for more balanced and integrated risk management frameworks that combine systemic governance, human-centered interventions, advanced engineering controls, and proactive environmental hazard management. Such integration is essential to address the complex, multi-layered risk landscape inherent in coal logistics operations.

### 4.4 Critical synthesis: reframing human error within a systemic context

A synthesis of the hazard analysis and risk management practices reveals a critical paradox within the occupational health and safety (OHS) landscape of coal logistics. While the thematic analysis identifies ‘Human and Organizational Risk Factors’ as the predominant hazard category (51%) and ‘Human Factors and Behavioral Control’ as a leading management strategy (70.6%), attributing accidents solely to individual negligence represents a reductive interpretation of workplace safety. It is essential to recognize that administrative procedures often provide only modest levels of safety performance. The persistence of poor procedures and inherent biases in investigations frequently hinder effective risk mitigation, demonstrating that procedural compliance alone is insufficient to prevent complex logistical accidents (20, 21).

The high prevalence of “unsafe acts” and “violations” reported in the reviewed literature (28, 31) should not be viewed merely as isolated behavioral failures to be corrected through stricter disciplinary mechanisms. Instead, viewed through a systemic OHS lens, these human errors are often “active failures” precipitated by “latent conditions” within the socio-technical environment of coal logistics. However, as Kletz (26) argues, listing “human error” as a root cause is far too vague to be useful because different types of error require entirely different preventive actions. Safety management must move beyond surface level mistakes and address the underlying causes of these infringements. The International Association of Oil and Gas Producers (32) emphasizes that context drives behavior, and research indicates that most safety violations are situational and unintentional rather than for personal benefit (33, 34). Frequently, employees breach rules because they believe they are helping the organization maintain operational efficiency under unsustainable demands (28, 31, 34, 35).

For instance, the established correlation between high workload, fatigue, and “unsafe worker states” (35) suggests that many errors are rational biological and psychological responses to unsustainable operational demands and psychosocial stress, rather than issues that can be resolved by behavioral coercion alone (35). Therefore, current risk management practices that rely heavily on punitive behavioral compliance, such as the “three disobeying” punishment systems widely cited in the literature, address the symptoms rather than the root causes of occupational hazards.

These punitive methods contrast sharply with the five human performance principles established by the International Association of Oil and Gas Producers (32). These principles assert that error is normal, blame fixes nothing, context drives behavior, learning is vital, and how leaders respond matters. To advance safety performance and protect worker well-being in coal logistics, there is a compelling need to shift from a “blame culture” to a holistic “systems approach” aligned with these principles. This entails prioritizing engineering controls, workload optimization, and proactive psychosocial risk management over administrative sanctions, ensuring that the OHS framework promotes systemic resilience even when human error inevitably occurs.

### 4.5 Limitations of the study

While this systematic review rigorously applied the PRISMA 2020 framework (22) to analyze systemic risks, several limitations must be acknowledged. First, the search strategy, although robust, may not have captured all relevant literature. Future studies should consider broadening the keyword coverage to include terms such as “supply chain safety,” “maritime safety,” or “freight logistics.” The absence of these specific overlapping terminologies in our current search strategy means that some highly relevant studies, particularly those embedded within broader supply chain management or maritime port literature, may not have been fully captured in this synthesis. Second, the restriction to English-language publications indexed exclusively in Scopus and Web of Science may introduce language and database publication bias (24). Finally, the overrepresentation of studies from certain regions, particularly China, may limit the direct applicability of the identified organizational safety cultures to other global contexts. Future research must evaluate how variations in safety standards across different countries affect the generalizability of these systemic interventions before they can be universally implemented.

## 5 Conclusion

This systematic literature review has synthesized findings from 68 peer-reviewed studies published between 2004 and 2025 to identify key occupational accident risks and management practices within the specific domain of coal logistics. Unlike previous reviews that conflate logistics with general mining extraction, this study isolated the post-extraction supply chain encompassing transportation, handling, and storage, to provide a targeted understanding of its unique risk profile.

The thematic analysis reveals that while Human and Organizational Risk Factors constitute the predominant hazard category (51%), a critical interpretation suggests that “human error” is frequently a symptom of deeper systemic deficiencies rather than the root cause. The high prevalence of unsafe acts and violations reported in the literature often reflects “active failures” triggered by latent organizational conditions, such as unsustainable workloads, fatigue, and production pressure. Consequently, the industry’s heavy reliance on behavioral control strategies (70.6%) and punitive disciplinary mechanisms may be insufficient if the underlying work systems remain flawed.

Mechanical and operational failures, particularly conveyor belt deviations and dynamic load risks in heavy haulage, represent the second major risk category. These technical hazards are shown to be universal across different geographical contexts, governed by physical laws rather than regulatory boundaries. However, the management strategies identified, specifically the top-down Safety Management Systems and state-led intelligent automation initiatives are heavily influenced by the Chinese context, which accounts for 66% of the reviewed literature. While the technical validity of these findings is robust globally, the organizational applicability of strict command-and-control safety models may require adaptation when applied to different cultural or regulatory environments.

In conclusion, effective accident prevention in coal logistics requires a paradigm shift from a “blame culture” focused on correcting worker behavior to a “systems approach” that addresses latent organizational hazards. Future research should prioritize: (1) cross-national comparative studies to test the generalizability of safety management frameworks outside of China; (2) the integration of real-time environmental monitoring to manage dynamic risks like spontaneous combustion; and (3) empirical evaluations of holistic interventions that combine engineering controls with psychosocial risk management. This integrated approach is essential to build resilience within the complex, high-risk environment of coal logistics operations.

## Data Availability

The original contributions presented in the study are included in the article/supplementary material, further inquiries can be directed to the corresponding author/s.
